# Mr. Cross on the Varioloid Epidemic of Norwich, with a General Exposition of Varioloid Diseases

**Published:** 1820-09-01

**Authors:** 


*A History of the Variolous Epidemic, which occurred in Norwich in the Year* 1819, *and destroyed* 530 *Individuals; with an Estimate of the Protection afforded by Vaccination, and a Review of past and present Opinions upon Chicken-Pox and modified Small-Pox.* By JOHN CROSS, Member of the Royal College of Surgeons in London, Corresponding Member of the Societé Médicale d'Emulation of Paris, late Demonstrator of Anatomy in the University of Dublin, &c. 1 vol. Octavo, pp. 296. London, 1820.

To restrain within wholesome limits an increasing population, it hath pleased Divine Wisdom to visit mankind, from time to time, with one or more of those savage destroyers of the human race; namely, war, pestilence, or famine. Terrible as these evils will appear at the moment, there is no doubt that they are ultimately designed for our benefit.

"A human form, pampered, bloated, and plethoric, will often have the appearance of strength as well as magnitude; though no state of it can be less adapted to facilitate the animal movements, or in greater danger of a hasty dissolution. The body politic also loses in muscular force, as much as it acquires of unwieldy size, till, by the gradual decrease of vigour and augmentation of weight, it totters on its baseless supports, and, at last, lies level in the dust with Babylon and ancient Rome."*

Although it may never be intended for human skill to annihilate the fell monster, which forms the subject of the work before us; yet, an experience of more than twenty years has shewn, that the great Jennerian discovery has the power to disarm it of its violence, and to regulate its course, so as to render it comparatively innocuous. We may guard against the furious blast or the raging storm; but who can stay their approach, or control their duration, except the

---

* Essays, Moral and Literary, by VICESIMUS KNOX, D. D.

sublime Author of Nature, who unfolds the elements and agitates the Universe?

During a period of national adversity, various moral, as well as physical causes, which we need not detail, conspire to assist the generation, and to extend the ravages of epidemic diseases. Thus, the animal spirits droop, and the sanguineous stream, abating in its wonted force, deserts the smaller passages, and presses around the great vital organs. That fine harmony, which should animate every fibre, is disturbed; the nice balance of the moving powers is destroyed; and the first impression of morbific causes benumbs and prostrates the enfeebled and starving multitude.

Mr. Cross has divided his work into two parts. In Chapter I, which treats of the small-pox, he observes, that Norwich has long been celebrated for the salubrity of its atmosphere, and the great age to which a considerable portion of its inhabitants has attained. Its buildings are spread over an extensive surface of ground; its population, amounting to about 40,000, one third of whom, employed in trade and manufactures, is generally characterized by cleanliness and industry. Hence it has been comparatively exempt from contagious and epidemic diseases. In 1805 the small-pox prevailed so much as to excite some attention; and a second time, in 1807, when it became so extensive as to occasion 203 deaths to be recorded in the bills of mortality, between the latter period and the year 1809. It appeared again in 1813, when 65 deaths took place. It was accidentally introduced in 1818; and in the latter end of February, 1819, it extended from one of the charity-schools to all quarters of the city. From the list of burials, taken from the bills of mortality, it appears, that the greatest number of deaths took place during the months of June and July; the average being about 150 in each of those months. Of the children attacked, under 2 years of age, 260 died; under 4 years, 132. From this age onwards, the fatality progressively diminished, and no death is recorded of any person beyond the age of 40. The epidemic was confined almost exclusively to the lowest orders of the people, of whom about one in six affected with it died. Considerable variety was observed in the features of the disease; and all the petechial, and most of the confluent cases, terminated fatally.

"In one child mortification commenced in the cheek, and extended over one half of the face. In a girl, 14 years of age, who ultimately died, the labia pudendi sloughed away; a phagædenic ulcer, in a third case, spread from the angle of the mouth and destroyed the lower lip."

Bloody stools attended many of the worst cases; but the urine was not observed to be tinged with blood. The prejudiced and ignorant being the principal sufferers, the prescriptions of old women were more listened too than the advice of the medical attendant. Hence the phlogistic treatment prevailed, often to an injurious extent.

"In singulis ferme ædibus reperiatur stolida aliqua, ac sciola, mulicrcula, quæ in hominum perniciem, quam non didicit, artem exerceat."*

Several instances of severe small-pox occurred in adults, who had, at various times before, resisted the most intimate and continued exposure to variolous contagion. The following fact was related, by one of the Suttons, to Mr. Bayley.

"A man, who believed himself to have had the small-pox, lived for twelve years as a nurse in the establishment for the reception of innoculated patients, which the Suttons had near Norwich, continually waiting upon the patients, who were undergoing the disease, and at the end of that time he caught the small-pox, of which he died."

A few instances occurred, in which there was reason to believe, that the disease had twice happened to the same individual, with the interval of several years between the two attacks; but our author only saw one well-attested case of small-pox after inoculation.

### Chap. II. *Of the Cow-Pox.*

The neglect of vaccination, which we lament to say has of late prevailed in all parts of the kingdom, was no less remarkable at Norwich; particularly among the lower orders of the people. So great, indeed, was the reluctance of the poor to accept gratuitous vaccination in that city, that Dr. Rigby suggested to the Court of Guardians, the expediency of allowing half-a-crown to every poor person, who should bring a certificate from a surgeon of having satisfactorily gone through the cow-pox. This plan having been adopted, 1066 persons claimed and received the donation, between the 12th of August and the end of December.† It has been customary for the vulgar, in all ages, to abuse their prosperity, and, forgetting the author of it, to neglect those means, whereby they might merit its continuance, until the day of adversity is at hand. This is beautifully described in the divine song of Moses, addressed to the Israelites in the following words:

"Thou art waxen fat, thou art grown thick, thou art covered with fatness; then he forsook *God which made him, and lightly esteemed the Rock of his Salvation*."‡

The humane plan of Dr. Rigby having been found ineffectual, and 1700 persons still remaining liable to the attack of small-pox, it was agreed, by the medical men in Norwich, that vaccination should not only be practised by them gratuitously, but that they should attend on the poor at their own habitations. This philanthropic conduct was deserving of the highest praise, particularly as

---

* Sydenham: Opera, p. 153.

† See Dr. Rigby's Appendix to Further Facts relating to the Case of the Poor, &c.

‡ Deuteronomy, Chap. xxxii. verse 15.

it was pursued in a time of public distress, and, in some instances, with inconvenience and hazard to themselves. We regret to add, that it did not fully answer their expectation, and to acknowledge, that we ourselves have often been induced to take the same step, and, after much persuasion and repeated visits, have only succeeded to a very limited extent. The number of persons vaccinated by our author exceeds 700. In every case he made two incisions in each arm, and employed ichor taken on the eighth day. He found about one in 50 incapable of taking the disease; and out of 500, of whom he kept a regular account, only 384 had it in a satisfactory manner. One child took the complaint after having been punctured in four places at six different times, and one or two resisted it in consequence of a cutaneous eruption. Of 112 families, comprising 603 persons, whom the author attended, and of whom he kept a regular register, "200 had small-pox."

One of these "had three months previously gone through the chicken-pox, and another had the same disease a few weeks after the small-pox; 91 had been vaccinated, two of whom had modified small-pox, and one had chicken-pox; and the remaining 312, who had small-pox formerly, or possessed a peculiarity of system, enabling them to resist it, had no eruptive disease of any sort during the epidemic."

On inquiring into the practice of others, the author observes, in a note, that he found only 11 out of 420, who had formerly been vaccinated, had suffered from an eruptive disease during the epidemic. These were all unattended with danger, and seemed to have been instances of modified small-pox. Besides the exposure to variolous contagion, several hundreds of those vaccinated from the earliest period of the practice, until within a few weeks, have been subjected to the additional test of inoculation with variolous matter during the epidemic, and in no instance has *regular small-pox*, as far as the the author has been able to ascertain, been produced.

Chap. III. *Of Small-Pox and Cow-Pox occurring together in the same Individual.*

The simultaneous appearance of two constitutional diseases in the same individual is a rare occurrence. It is, however, an established fact, that the cow-pox and small-pox are occasionally found existing together. The period at which the latter may present itself after vaccination is uncertain; it has been noticed as late as the thirteenth or fifteenth day, generally observing an imperfect course; but most writers in Britain seem to regard the fifth or sixth day as the latest time, at which it may appear and go through a severe course in conjunction with cow-pox.* In his popular View of Vaccine Inoculation, Dr. Adams says, "that when the feverish disposition, aris-

* George Bell on the Cow-Pox, p. 81.—Willan on Vaccine Inoculation, p. 7; and his Reports on Diseases of London, p. 314.

ing from natural small-pox, shews itself on the third day of vaccination, the latter will rather be hastened in its progress. It will afterwards proceed with the small-pox-pustule, retaining its proper figure, but without that surrounding redness, which marks its genuine character. Although the eruption of small-pox may come out during the progress of cow-pox, the contrary does not take place; when the former has come out on the second or third day after the insertion of vaccine virus, the latter has rarely produced any effect. In general, the later the period at which the small-pox has appeared, the more mild has it been rendered, and the more perfectly have the characters of the vaccine disease been maintained." When the variolous eruptions have appeared later than the ninth or tenth day after vaccination, our author has found them mild in their character, small in their number, and lasting only three or four days. A few exceptions to these general remarks are described in four cases, for the particulars of which we must refer our readers to the work itself. The following rules of practice, drawn from his observation and experience, we shall give in Mr. Cross' own words.

"*Firstly*, to vaccinate, although the variolous contagion may already have been caught, as the small-pox, if not prevented, is most likely to be mitigated, should the vaccination take effect; *secondly*, to avoid all unnecessary exposure of patients, undergoing the cow-pox, to the variolous contagion, until the areola is fading, because, although a great proportion will escape, there is a chance of some being affected by it, and it is desirable to shun even the most mitigated form of the variolous eruption; *thirdly*, when small-pox is prevailing, to give no decisive opinion of protection by cow-pox until the scab is about to fall off, that parents may not be deceived, nor the powerful means of safety be unjustly brought into discredit." *Chap. iii.*

From the existence of the two diseases together, it must not be understood that each maintains its usual intensity, and observes its regular course; on the contrary, it will be found that one of them will be attended and mitigated by the other, according to circumstances.

### Chap. IV. *Of Small-Pox after Cow-Pox.*

Our author's experience has not furnished him with a single instance of *regular* small-pox after cow-pox; and after the most diligent inquiry, he has not met with more than five cases in the practice of his professional brethren. Three of the patients recovered; the others died, one of them having confluent, and the other petechial small-pox. These failures can have no weight against the practice of vaccination, when compared with the immensity of 10,000 vaccinated individuals living in the midst of a contaminated atmosphere; while no less than 530 deaths were recorded out of little more than 3000, who had neglected to be vaccinated.

### Chap. V. *Of the Concomitant Eruptive Diseases of the Epidemic Small-Pox.*

The measles, though frequent and fatal the preceding year, were hardly met with in 1819; the hooping cough often occurred; the scarlet fever, in a few instances, appeared; but, on the whole, the contagious complaints incident to children were less frequent during the epidemic. The author, however, has confined his observations to those complaints which seemed to him to be directly influenced or produced by small-pox; and of these, the first he has noticed is *petechial fever.* It attacked only a small proportion of those liable to small-pox, and generally proved fatal; and during the whole of the summer no instance of it was seen in those who had previously gone through small-pox or cow-pox. The victims of it were chiefly feeble children. After symptoms of previous debility, a few purple or dark spots appeared, increasing in number daily, occupying the principal parts of the body, and varying from the size of a pin's head to that of a kidney bean. The colour and shape of the spots mostly resembled those which might be produced by scattering over the body ink from a writing pen. Every case seen by Mr. Cross terminated fatally, from the third to the tenth day of the eruption. In a petechial cadaver, examined by himself and Dr. Yelloly, the purple spots were found to penetrate into the substance of the temporal muscle. The stomach was contracted in the middle, like an hour-glass, and contained half a pint of dark, green, ropy fluid. Its inner surface was beset with petechial spots, like the external skin; yet there were no petechiæ in the lining membrane of the œsophagus, nor of the large and small intestines. The patient died on the fourth or fifth day of the eruption.

"Mr. Hull dissected a child a year and a half old, which (who) died on the sixth day of the variolous eruption, and found the rectum, colon, and ileum, marked with circular patches, distinct, white, and having an appearance of indentation in the centre. They were here and there congregated into clusters, and were situated beneath the mucous coat, resembling in figure and diameter of surface the cutaneous eruption, of which they seemed, as Mr. Hull himself well expresses it, to be faint imitations. White patches were also observable in the jejunum; but neither circular nor indented. The œsophagus, stomach, and duodenum, presented none of these appearances."

The next eruptive disease enumerated by our author, occurred indiscriminately in those who had passed through small-pox and cow-pox, and in those who had not had either of these disorders. Febrile symptoms having existed a few days, numerous vesicles broke out, transparent as crystal, and filled with a colourless fluid, enveloped in the delicate cuticular membrane on the shoulders, arms, face, and body. Many were large, oval, and prominent; others were irregular and jagged at the edges; while some were as small as pins' heads, and pointed at the top. They were surrounded with an irregular blush, and existed in considerable numbers on the scalp. Intolerable itching was present. About the third day the cuticular covering became semi-opaque, and its contents turbid or milky. Until the fifth or sixth day a succession of crops came out, and a few days afterwards the crusts fell off, leaving irregular stains upon the skin. The throat was usually sore, and every vesicle consisted of a single cell, which the author believes to be a characteristic mark of the eruption. In the decline of the disease, the contents of the vesicles approached to pus. The eruption was contagious, and was never observed to be followed by small-pox, although there was reason to believe that such an event occurred. Two children, however, were vaccinated after its occurrence with perfect success.

The last section exhibits the eruptive diseases, which had *chiefly* occurred in those who had passed through the cow-pox. Some of them consisted of a rash with sore throat; others of a conoidal, vesicular eruption; but the most important cutaneous affection presented pocks of the indented character, the different varieties of which are illustrated by numerous cases, which our limits will not permit us to transcribe. These indented eruptions were commonly preceded by vomiting, restlessness, occasional delirium, and other symptoms of fever, which was generally of the inflammatory, sometimes of the bilious, and in a case or two of the typhoid kind.

"The first appearances of the eruption have been small red specks, in the centres of some of which minute circular pocks, remarkably indented in the centre, have been evident in twenty-four or thirty-six hours. As to number, they have varied from twenty or thirty to as many hundreds."

They have been found to advance most rapidly on the face, in which situation, the indented character has been lost on the third day, and on the fourth and fifth the pocks have been filled with a purulent fluid. Even when not much crowded, they have run into each other, attended with some swelling of the cheeks and eye-lids on the fourth or fifth day, but never so as to close the latter, and always forming crusts of an irregular shape, making rough and accumulated masses.

"On the hands and arms, the indented pocks have been most numerous, and have mostly lost the indentation on the fourth or fifth day, their surfaces changing from flat and depressed to convex or spherical, and their contents passing from a colourless lymph into a turbid, wheyey, muddy, and, in a few pocks, even purulent fluid. The largest distinct pocks, examined at this time, have exhibited the base occupied by adhering lymph, which could not easily be removed. The pocks in this situation were hard and resisting to the touch, never being broken by accident, and never marked by a red circle at their circumference, save a minute line, to be discovered only by the magnifying glass. Upon the trunk the eruption has always advanced with less regularity, being often interspersed with irregular vesicles, consisting of one cavity, and forming no regular scabs. Similar appearances in the progress of the eruption have been visible on the lower extremities, a few only advancing so as to be indented and subsequently convex, whilst the rest have remained as conoidal vesicles or circular specks under the cuticle, forming no prominent eruption. Incrustation has been nearly completed, in most cases, by the sixth or seventh day."

On the upper extremities the incrustation was most perfect; every pock, which in its progress was indented, left a flat, circular, and solid scab, frequently marked by a depression in the centre, and seldom more than one-eighth of an inch in diameter.

In every instance the throat has been sore; and on the third or fourth day small circular yellow specks on the tonsils or posterior fauces were always to be found. The scabs, on falling off the cheeks, left tubercular elevations, which were absorbed in a week or two. The traces of the disease, after desquamation on the rest of the body, were confined to the temporary appearance of small, circular, dull, red specks; excepting the arms, which, for the most part, presented slightly convex surfaces, not distinguishable by the touch. No pits remained, excepting a few trifling ones upon the forehead, nose, or cheeks; neither was deformity, permanent injury, or death, produced in any instance. The mildest cases corresponded with certain descriptions of varicella, and the most severe were regarded as modified small-pox. The author, however, considers them all to be a series of the same complaint, and feels little hesitation in announcing his belief, that they proceeded from the variolous contagion. They occurred when small-pox was most prevalent, often when subjects suffering from it were in the same room; they were at other times traced to exposure to it, and at others they preceded for a few days the appearance of variola in the family. The cases, which never advanced so far as to present indented pocks, he has not known to be submitted to any experiment; but the mildest examples, where such indentation existed, were found communicable by inoculation, producing, in a few instances, small-pox in those who had not before had either that disease or cow-pox; while in a small proportion of the vaccinated, they produced an eruption similar to themselves in mildness and duration. These experiments were repeated by parents as well as medical men, and thus extended the opportunities of watching the results. The character which Mr. Cross regards as common to the whole of these eruptions is, that in each case some are found to consist of pocks, which are polyœcious or multi-cellular, which structure is proved to exist in all those possessing an indentation in the centre; and it is this cellular structure that distinguishes them, in the eye of the pathologist, from the vesicular eruption, described in the second section of this chapter.

### PART THE SECOND. CHAP. I. *Of the Distinctive Characters of the Small-Pox.*

When this disease prevails epidemically, it sometimes destroys above one third of those affected by it; and at others, not more than one out of fifty. From these extremes, it is evident it must be subject to great variety. In its regular course, it is preceded, even in mild cases, by three or four days of severe indisposition; and the manner in which the eruption commences has ever been regarded as characteristic. It appears generally first upon the face, is completed in three or four days; on the fifth or sixth day the contents of the pustules are changed to a turbid fluid; and on the eighth day they become distended with pus. Dr. Adams and Dr. Jenner have recorded a variety of the disease, which does not proceed to perfect maturation.

At the time of suppuration, a crimson areola encircles the variolous pustule, and the febrile symptoms return during the suppurating stage, and attend the commencement of scabbing. Where the eruption is plentiful, this secondary fever is always present; together with a peculiar fœtor.

The late Mr. John Hunter, in dissecting a fœtus born with the variolous eruption, found in each pock a slough of the cutis vera, answering in dimensions to the size of the pustule. This slough, in some instances, forms the pitting, and he believed it to be confined to the small-pox, and not so much owing to the intensity as to the peculiar kind of inflammation. Mr. Ring and Dr. Adams agree, that this slough forms the certain sign of variola. The pits which follow the complaint are various; and it has been asserted, that those which are irregular, and of the same colour with the surrounding skin, proceed from genuine small-pox; while those which are circular, superficial, large scars, of a lighter colour than the adjoining skin, are the consequence of a spurious disorder.

As we never wish to see small-pox inoculation revived, we abstain from following our author in his attempts to ascertain a criterion whereby we may decide its security; and hasten to another part of the subject, to which he appears to have paid great attention; namely, the anatomical structure of the pock. After animadverting on the arrangements of some of our most celebrated Nosologists, he observes, that the first indication of change in the cutis is the appearance of a small red spot, in the middle of which a firm knot is perceptible to the touch, although not so soon visible. In twenty-four hours, a pimple is observed in the centre, which increases so as to present an accuminated vesicle, which gradually changes its form, and on the fourth day becomes a vesicle perfectly circular, somewhat flattened on the top, and indented in the centre, as if the point of a pin had been pressed upon it, and had left the impression. The vesicle is now one eighth of an inch in diameter, and has often a reddish or bluish appearance, proceeding from the inflamed and vascular portion of the skin being seen through it and through a limpid fluid, which is contained in different cells. To a cellular structure of this sort, the author thinks the term *pock* should be confined. The walls of the cells being perfectly transparent, their disposition during life is not easily ascertained. The arrangement of the partitions first described to him, by Professor Macartny, of Dublin, he has found, after death, to correspond with the axis, spokes, and circumference of a wheel. The vaccine pock bears a strong resemblance to the variolous, in being multi-capsular. Dr. Willan, speaking of the cells of cow-pox, says, "these are perhaps only a portion of the *cellular membrane* distended by the effusion of lymph." Mr. Cross justly censures this opinion; and observes, that the cellular membrane is out of the way: the dense and less vascular part of the skin being between it and the basis of the pock.

The cellular structure is formed by the *rete mucosum*, or by a freshly organized substance thrown out by the cutis itself, and excavated into cells for holding the fluid of the pock. The walls of these cells secrete the contained fluid, and if this be partly let out by a puncture, the drying of the lymph closes the part opened, and the fluid being again secreted, distends the pock to its former shape. The variolous pock grows more in circumference than elevation after the third or fourth day, and on the fifth or sixth its size is a quarter of an inch, when the indentation is less observable, and the contents cease to be transparent. A red circle shows itself at the circumference, and becomes wider as the pock increases. After this, the surface becomes convex, the point, which was indented, being now the highest part; the circular shape is exchanged for one oval, oblong, or irregular; the contained fluid, at first turbid, is afterwards purulent, and the cellular structure is altered, the walls or partitions being thinner, broken up, or partly absorbed, so that a great proportion of the fluid will escape by one puncture. These changes are effected by the eighth day, when incrustation commences.

In the most regular small-pox, the eruption advances variously, being influenced by the structure of the parts affected; and mild cases may occur, which cannot, by any single rule, be determined to be variola. Even the existence of a slough deserves not to be implicitly relied upon: its presence in all the pocks only proving small-pox; but its absence, from a great proportion of them, not proving the contrary.

### Chap. II. *A Sketch of the History of Varicella.*

An imperfect eruption, not protecting against a future attack of small-pox, was noticed as early as A. D. 900, by Rhazes; and since that time by Vidus Vidius, Sennertus, and Riverius. It was variously denominated: *e. g.* spurious, lymphatic, crystalline, &c. Sydenham, too, alludes to a spurious kind of small-pox, having no connexion with the real disease; and about 1690, Morton adopted the term *chicken-pox* from the vulgar, and introduced it into our Medical Nomenclature. At this period, some regarded the eruption as the mildest small-pox, while others believed it to be of a different genus; but they all agreed, that it had no power to prevent small-pox.

The variolous inoculation, which was introduced into England in 1722, may be considered as a second æra in the history of varicella. The principal writers on this subject were Fuller, Hoffman, and Van Swieten. The last author divided the spurious pocks into three species: the *steen-pocken*, *the water-pocken*, and the *wind-pocken*. Vogel accepted the term varicella; and Sauvages remarks, that "what the English call the *chicken-pox* is that variety of small-pox, in which the pustules terminate by resolution on the seventh day, without suppuration or any perceptible fever, and without danger." In the year 1767, Dr. Heberden read a paper on chicken-pox. It does not appear that he ever witnessed inoculation from this disease, but he implies, that it can thus be communicated. Dr. Sims' account of chicken-pox, in his Treatise upon Epidemic Diseases, is unfortunately rendered imperfect by his omitting to mention whether the small-pox happened to be prevailing at the same time. Hence we are uncertain whether the complaint described by him was spurious small-pox, or a mild sort, vulgarly denominated *swine-pox*, and mentioned by Dr. Jenner, as we have before remarked. John Hunter and Dr. Adams observed, that chicken-pox does not commonly produce a slough. Cullen made it a distinct species.

The discovery of the immortal Jenner, which was made known in 1798, forms a third period in the account of varicella. Dr. Frank, of Vienna, separated it entirely from variola, and placed it in a different genus, under the title of *Pemphigus Variolodes*, which he subdivided into *vesicularis* and *solidescens*. Mr. Ring related a case of *confluent* varicella, and observed, that the chicken-pox occasionally extends to the fourteenth day. Dr. Willan, who certainly formed the best arrangement of diseases of the skin, which was ever published, divided the varicellous eruptions into three species; the *lenticular*, the *conoidal*, and the *globate*. As he no where represents these pocks to be evidently indented upon the surface, it is probable that he considered all eruptions, having that character, as modified small-pox; as did also Professor Montesante of Padua, who published a memoir on the disease in 1816. When the small-pox prevailed epidemically at Montpelier, Berard, and De Lavit believed it to have the same origin with varicella, which occurred at the same time, while Dr. Fontaneilles, who practised not fifty miles from that place, referred every example of chicken-pox to a separate and distinct contagion. It was even propagated from one individual to another, in the latter instance. Moore states, that on the first day a small vesicle may usually be seen, and little or no coagulable lymph is effused to thicken the cuticular membranes; a serous secretion is poured out under the *rete mucosum* almost with the rapidity of a blister, and little vesicles are quickly formed, more pellucid than those in small-pox, yet surrounded with inflamed borders of various breadths. "On the second day the vesicles are larger, but neither concave, clouded, nor hedged round with coagulable lymph. But as they fill, the liquid separates the *rete mucosum* and cuticle from the cutis; and as one side yields more than another, the vesicles occasionally become oval, or lenticular, or in some degree irregular. Often, however, they retain a regular round figure, but not so constantly as in small-pox." Professor Monro believes the disease to proceed from a distinct contagion, to occur only once to the same person, and to be incommunicable by inoculation. Mr. Bryce, with most English writers of the present time, considers it to be a disease, *sui generis;* and his description of it is corroborated by additional remarks of Dr. Abercrombie and Dr. Alison. We have lately noticed a distinctive mark, related, we think, by Dr. Thompson of Edinburgh, whereby our diagnosis is likely to be greatly assisted. On breaking the vesicle or pustule of variola, by rubbing the finger over it, a *tubercle* is found, which is not perceptible in varicella. This tubercle is occasioned by a peculiar inflammation of the true skin, and is probably the rudiment of the subsequent slough.

### Chap. III. *Of modified Small-Pox, and its Classification with Variola and Varicella.*

Soon after the introduction of cow-pox, it was ascertained that the variolous contagion was capable, in some instances, of producing the same effects at a remote period, as during the progress of the vaccine disease, giving rise to a mitigated variolous eruption, which Mr. Dunning, one of the earliest supporters of Vaccination, denominated *modified small-pox*. In France, where the small-pox has lately existed epidemically, a violent controversy arose among the medical men respecting the eruptive disease, which appeared in those who had been vaccinated; some giving it the name of modified small-pox, while others as strenuously believed it to be a severe kind of varicella. In Holland, the same appearances presented themselves; and in Wertemburg, where the epidemic also prevailed, the vaccinated were found to escape with a mitigated variola, excepting two instances, in which the disease proved fatal.

In the eruptive complaint, which has been styled modified small-pox, the preceding symptoms resemble those of the regular small-pox, and are relieved on the appearance of the eruption, which is first observed on the hands or face, resembling red spots of inflamed cutis, and attended with thickening sufficient to be perceived by the touch. In twenty-four hours a pimple is to be distinguished, which soon enlarges into a conoidal vesicle, seated on a slightly elevated basis; and on the third day circular pocks, indented in the centre, firm and resisting, are intermixed with others less advanced. On the fourth day some become convex, and resemble small pearls, while others remain indented. The larger ones can now be ascertained to be cellular, and contain a clear fluid at the summit, and a firm lymph at the basis; and if the whole be taken away, the cutis beneath presents a convex surface. Those on the face contain a bright yellow fluid, and the largest, in other parts of the body, are filled with a milky, or thick dirty liquor. Without any return of fever, the incrustation commences on the third, fourth, or fifth day, and is nearly completed by the sixth: many of the scabs being solid and circular, and the largest not often exceeding one-eighth of an inch in diameter. The throat, during the eruption, is sore; and on the second or third day, the tonsils and posterior fauces almost constantly exhibit yellow circular specks. At the end of three or four days the scabs fall off, and leave tubercular elevations, particularly on the face and arms. As the central depression disappears in the progress of the complaint, the eruption should be carefully examined during the first four or five days. When the indentation is observed, all agree that it is most marked, while the contained fluid is clear, and must not be confounded with the depression produced in other eruptions.

When the eruption is extensive, it may be attended with typhoid symptoms, but the secondary fever is entirely absent. In these cases the incrustation commences on the fifth day, and the declining period presents a variety of blotches, which never become prominent; pocks incrusted, pimples subsiding without crust, and a few pustules still distended with fluid until the eighth or tenth day. The flat scabs may remain for two or three weeks on the extremities. In rare instances, such cases have been complicated with petechiæ; and Moore observed a few cases, "in which the eruption was *confluent*, and did not reach the acmé until the eighth day. But even in these cases, no secondary fever ensued, and all the symptoms and vestiges of the disease vanished with remarkable rapidity, leaving few or no pits."*

"A rash occasionally precedes or accompanies the most regular cases of modified small-pox; and the mildest effect of variolous contagion (our author observes) "seems to be shown in the production of a rash and sore throat, without any eruption of vesicles or of febrile symptoms for a day or two, that subside without the appearance of any affection of the cuticular surface."

All these varieties are found to proceed from one source, namely, the variolous contagion; and the modified small-pox occurs mostly in those who are, by some natural or artificial protection, rendered incapable of going through the regular small-pox. The contagion from mild cases of the modified disease, acting on a person who has neither had cow-pox nor small-pox, will sometimes produce the latter with all its horrors; but its effects are better ascertained by inoculation. These experiments produce an extraordinary phenomenon, by inducing a disease differing both in character and severity from the one whence it was derived. In a small proportion of the vaccinated, inoculation will produce the modified disease; but it has not been ascertained how it will affect those who have had the small-pox. The experiment having been made on those who had neither cow-pox nor small pox, it has been found that a vesicle on the arm, with a rash, or a few pimples on the body only, will sometimes be produced, leaving the subject equally liable to small pox.

The proportion of the vaccinated who receive modified small-pox has been variously stated. Our author's opinion is, that not more than one in twenty will be in any way affected by the most intimate exposure to variola in the same room; and that less than one in fifty will have the disease in a form answering to the generally received description of modified small-pox.

Having considered and described the eruptive diseases following vaccination, we shall direct our inquiry into the causes of their

* Moore's History of Vaccination, p. 107.

happening to some and not to others. At an early period, Jenner observed, that the "constitution loses its susceptibility to small-pox contagion, and its capability of producing the disease in its perfect and ordinary state, in proportion to the degree of perfection, which the vaccine pustule has put on in its progress; and, that the small-pox, if taken subsequently, is *modified* accordingly." This, like most of his opinions, has been supported by the testimony of subsequent experience; and incomplete vaccination having not improperly been considered as one cause of failure, the propriety of leaving one vesicle untouched has been suggested, and we believe the practice has been of late extensively adopted. Diseases pre-occupying the system or the surface of the cutis, eruptions, scald-head, teething, prevailing contagious disease, are also enumerated by our author among the causes, which may interfere with the progress of the vaccine pock. Hodenpyl believes scabies, rickets, scrophula, and cancer, to have the same effect; and Elsässer mentions, that in a district, where scabies was endemic, many cases of regular, as well as modified small-pox, occurred among the vaccinated; and on inquiry, it was discovered that a scabious child had been vaccinated, from whom ichor had been taken, which produced an irregular and imperfect disease. Vaccination having been followed by small-pox to an alarming and fatal extent, in a district of Silesia, an investigation of the cause of failure was undertaken; and it was ascertained, that the people had all been vaccinated by the same surgeon, who had been in the habit of taking the virus as late as the *eleventh* day, often from vesicles, which had been rubbed or scratched, so as to be injured in their structure; and had even raised an imperfect scab to obtain what moisture he could, with which he had propagated the disease.

With respect to the period at which the modified eruption may occur after vaccination, our author says it may happen at any time after the first few weeks; and that the former disease, in general, proves less mild in those above three or four years of age, than in those who are younger; and that it is rarely attended with danger at any age. Of Dr. Gibson's two hundred and fifty-one cases of *small-pox* after *vaccination*, it appears, that by far the greatest number attacked were those vaccinated less than two years. Dr. Thompson's varioloid eruptions occurred at various intervals, from a few days to fifteen years; not warranting the suspicion that the preventive or modifying power of the cow-pox was weakened or exhausted by time: increasing years appearing, in general, to lessen the susceptibility to small-pox contagion.

When the local and constitutional effects of the vaccine disease have been apparently perfect and regular, small-pox will now and then follow. This, it is evident, must be owing to peculiarity of constitution, as the same occasionally happens after variolous inoculation.

The modified small-pock resembles the chicken-pox, in consequence of its only passing through the papular and vesicular stages; but a little attention will enable the practitioner to distinguish them.

The former may also be recognized, when small-pox are suspected, by observing some vesicles within twenty-four hours after the first appearance of the papulæ; that the largest pox seldom exceed a quarter of an inch in diameter; that the size of the scabs bears a proportion to them; that the scabbing process is accomplished at different periods, from three to eight days; and that *a few of the pocks only advance to an imperfect suppuration*, the rest being checked in their progress, remaining as spots or pimples, or incrusting *while their contents are transparent.*

The author believes the term varicella to be strictly applicable to the modified small-pox; for which opinion he offers the following reasons. All the forms of varicella have, at various times, been classed as small-pox; and *vice versa.* In those parts of the continent, where this disease exhibited indented pocks, modified small-pox has been very rare. The mildest cases of modified small-pox are not incompatible with the descriptions of chicken-pox, by the most esteemed modern authors in England. To complete the analogy, he proposes to divide the genus into two species, or the species into two varieties:—1. Varicella *cellulosa*, or *cellular chicken-pox*, the synonymes of which are *stone-pock*, *horn-pock*, *modified small-pox*, *pemphigus variolodes solidescens.* 2. Varicella bullosa or vesicular chicken-pox, the synonymes of which are *crystals*, *water-pox*, *pemphigus variolodes vesicularis*, *mild vesicular small-pox.* As the titles sufficiently indicate the structural difference of the two species, we shall not detain our readers by any farther explanation.

### CHAP. IV. *Of Variolous Inoculation, and the Means of discouraging it.*

During the first eight years after the introduction of this practice into England, only eight hundred and ninety-seven individuals submitted to the operation, of whom seventeen died. Its adoption in other countries was even slower and less extensive: in some, it was not employed for above half a century; and in others, from its disasterous effects in spreading the contagion, it was actually prohibited by an act of the legislature. Dangers, as well as difficulties, were found to accompany it, and the number of deaths has been rather increased than diminished by it. This fact has been recently illustrated by Sir Gilbert Blane in a paper published in the Medico-Chirurgical Transactions, Vol. x. Part 2.

Respecting the small-pox hospital in this metropolis, our author says—"Inoculation is still performed upon those who choose to be admitted for the purpose, but the number has been small, six hundred and ninety-one only having been inoculated as in-patients from May 1808 to December 1819; of these, thirty-four were inoculated in 1818, and forty-four in 1819. Although the amount is so trifling, it must remain a subject of regret, that a practice discouraged by every associated body of medical men in the universe, and prohibited, under severe penalties, in half the nations of Europe, should be apparently sanctioned by a charitable Institution, originally established with the most humane views, and supported by individuals of the greatest benevolence. I have been informed of the small-pox being twice brought into the neighbourhood of Norwich, by patients discharged from this Institution, the contagion being probably conveyed by their woollen-apparel, which had undergone no purification previous to their dismissal."

From the opening of this Institution to the end of 1819, including a period of seventy three years, the average number annually inoculated was six hundred and fifty-eight; while, during a period of twenty one years from the introduction of cow pox to the end of 1819, the average number vaccinated was 2224. Dr. Bremer has arranged a table of deaths from small-pox in Berlin, before and since the practice of vaccination, from which it appears that the numbers sacrificed to the former were:

During inoculation, from 1790 to 1799 - - - 4117.
1808 — 1817 - - - 1367.

Here is a diminution of 2750 in the number of deaths during the latter period.

The want of some *efficient* means of preventing variolous inoculation and its consequences was severely felt at Norwich, during the season of the epidemic. Those labouring under small-pox exposed themselves in the streets, and on the public roads, at all times of the day; and itinerant inoculators, irregular practitioners, and old women, introduced and extended the disease to all quarters by inoculation, regardless of the admonitions given to them; because the law authorized no *direct* measures against them. To remedy these evils, our author proposes, with his accustomed humanity, several regulations, which we should be happy to see universally adopted; but we much fear that some of our wiser senators would forsee the ignorant opposition and danger which would arise, were any attempt made to diminish the *rights* and *liberties* of a British subject, except the whole nation were composed of *rational* beings.

### CHAP. V. *Of Vaccination, and the Means of promoting it.*

The progress of the genuine vaccine pock has been so well and so extensively described and understood, that a farther account of it would be superfluous. When the vesicle has risen properly, the areola formed duly, and all the characteristic local appearances are present, it is supposed that the system has felt the influence of the topical disease, and will afterwards resist small-pox. The symptomatic fever is not always to be observed. Soreness in the axilla is an uncertain symptom. The inserting of ichor afresh on the fifth day, which gives rise to a pock of diminutive size, *incrusting as early as the original one*, is doubtless, when it succeeds, the best test we possess.

The circumstances, under which it is expedient to re-vaccinate, are the following: where the disease takes a spurious or irregular character; where the system was pre-occupied by any disease; where the same supervened during the progress of cow-pox; and where the scar left is feeble and indented. Ichor should be taken, while limpid, from a genuine vesicle on the eighth or ninth day, just before or during the formation of the areola; and the patient furnishing it ought to be free from cutaneous eruption and constitutional disorder. It should be used, if possible, within forty-eight hours. Our author thinks it will be found, in general, most convenient to receive it upon points of quill or ivory; which, after becoming dry, should be corked up in a small phial. The plan we generally adopt is to confine it between two plates of glass, in which situation we have often found it perfectly fluid at the end of twelve hours, and more concentrated and equally effectual as when taken fresh from the arm.

The most eligible time for the operation is from the third to the sixth month; and where many are vaccinated at the same time, the surgeon will find it convenient to keep a register of the progress of the disease. The plan of making four punctures, and of leaving at least one untouched, is, we suppose, now universally pursued. The opening every vesicle and pressing out their contents are strongly reprobated by the author, who believes this practice may be the cause of failure.

In support of the general utility of cow-pox, we may observe, that, although our belief of its *preventive* power has been diminishing for some time past, yet repeated experience has so fully convinced us of its *modifying* property, as to remove all doubt on that subject in our own minds; and we believe that all the experienced and intelligent members of the profession have arrived at the same conclusion.

By endowing an Institution in this metropolis for gratuitous vaccination, and for supplying lymph to applicants in all parts of the kingdom, free of expence, the British Government has made an effort to diffuse the benefits of vaccination, which is highly creditable. *By making application to this (the* NATIONAL VACCINE) ESTABLISHMENT, *in an enclosure addressed to the Secretary of State for the Home Department, lymph can be obtained* FREE OF ALL EXPENCE. The poor, our author remarks, should be vaccinated at the expence of the parish in which they *reside;* and, in many situations in the country, we have no doubt that such an arrange- would facilitate the extension of cow-pox more effectually than any other. Our medical friends in the country inform us, that they experience increasing difficulty in promoting the practice of gratuitous vaccination, in consequence of a prejudice the poor entertain, either with respect to the quality of the morbific fluid employed, or the views of the inoculator. In large towns, particularly where the greater part of the inhabitants is employed in manufactories, Institutions should be formed to promote the general diffusion of the cow-pox; and where great apathy or prejudice might exist among the lower orders, we think it might be useful to connect some immediate advantages with the practice. The plan of allowing a bounty on inoculation, must, in populous districts, be partial in its operation; and those only would accept it who were in extreme poverty and distress, on account of the prejudices above stated, which we apprehend would be found prevailing in all parts of the empire; and which, we fear, would rather be increased than diminished by the practice. Our author proposes, that all heads of families should refuse to take into their houses servants liable to small-pox; and he thinks that guardians of the poor would not exceed their duty, by inquiring of every pauper, who applies for relief, on account of an additional number of children, whether these have been vaccinated, and by giving them accordingly a liberal or rigid allowance. It would be advantageous, if it were practicable, to compel all the poor indiscriminately, who have not had small-pox, to be vaccinated, before they receive parish relief. Their interests and immediate wants would thus invite them to perform their duty towards their children, without increasing the heavy burthens of the more opulent and industrious. A wholesome regulation had existed at Norwich, but was suffered to fall into neglect, which we hope will be put in force in our National Schools. It was a standing rule in a large establishment of this kind at Norwich, to admit none but those who had gone through small-pox, or been vaccinated.

In despotic governments the extent to which vaccination has been carried is astonishing. In Russia, no less than 1,200,000 received the benefit of it, between the years 1804 and 1812. In Denmark, the small-pox no longer exists; and in a *circular* addressed in July, 1816, to all magistrates and bishops in that country, it was ordered that all should be vaccinated, without a compliance with which injunction no individual could be received at confirmation, admitted into any school or public Institution, or bound apprentice to any trade. Priests were also forbidden to marry those who had not either had the small-pox or cow-pox. In Prussia, if any persons happened to die of small-pox, they were directed by an edict, published in 1816, to be buried within twenty-four hours, *silently* and *unattended*, without the tolling of a bell; and in such veneration is the great discoverer of vaccination held, that the 14th of May is made an annual festival to commemorate the day on which he made his first experiment. None but medical men regularly educated were allowed to vaccinate in the kingdom of Bavaria, and each was required to keep a register, which was returned to the government every three months. For this trouble they were rewarded according to the zeal they manifested in the cause. With the hope of wholly banishing the small-pox, it was enacted, by Maximilian Joseph, King of Bavaria, that from July, 1808, all persons above a certain age, who continued to neglect to be vaccinated, should be fined by an increasing penalty every year, so long as they refused to take the means for their own protection. Variolous inoculation was forbidden, and a penalty enforced against all those who performed or submitted to it. Measures equally coercive are now in force in the kingdom of Wirtemberg.

We have now presented our readers a full analysis of the work before us, with the exception of three appendices, the first containing a Translation of Dr. Heim's Paper on the Diagnosis between Variola and Varicella; the second, the Result of a Correspondence with Practitioners in the County of Norfolk, and adjoining parts of the County of Suffolk; and the third, an Abstract of a Report of the Vaccinations practised in France in 1816. The length to which we have already extended this article, will not permit us to enter into the particulars of these additamenta. We, therefore, refer our readers to the work itself, which we have no doubt they will peruse with as much pleasure and information as we ourselves have done; and we conceive, that all those who are not intimately acquainted with the latest information respecting the important subjects treated of in Mr. Cross's book, will do themselves an injury, if they fail to purchase it; and we earnestly recommend them to place it on the same shelf with the best dermalogical works of British and Foreign writers.

Mr. Cross had previously given a convincing earnest of his talent for observation, in his Medical Sketches of the Parisian Schools of Medicine; the present work realizes the expectations which we then formed of his professional character. The theatre for practical research and medical improvement in which Mr. Cross is now placed, will furnish him with ample materials for the exercise of that strong intellect which is lent to him, not solely for his own use, but also for the good of his brethren of the present and future generations.*

* We found it impossible to comprehend, in this article, an analysis of the very interesting and important work of Professor Thomson, of Edinburgh, on the varioloid diseases which have lately appeared in North Britain. We regret this the less, however, as the work itself, and a most valuable Review of it, in our highly respected cotemporary, the Edinburgh Medical and Surgical Journal for April last, have made the contents of the volume widely known throughout the profession. It appears, moreover, that Mr. Cross's work includes all that is known on the subject up to the present moment, besides several minute and important pathological particulars not hitherto ascertained.

